# Vitamin D Supplementation for Patients with Dry Eye Syndrome Refractory to Conventional Treatment

**DOI:** 10.1038/srep33083

**Published:** 2016-10-04

**Authors:** Seok Hyun Bae, Young Joo Shin, Ha Kyoung Kim, Joon Young Hyon, Won Ryang Wee, Shin Goo Park

**Affiliations:** 1 Department of Ophthalmology, Hallym University College of Medicine, Seoul, Republic of Korea; 2 Department of Ophthalmology, Seoul National University Bundang Hospital, Seongnam, Gyeonggi, Korea; 3 Department of Ophthalmology, Seoul National University College of Medicine, Seoul, Republic of Korea; 4 Department of Occupational and Environmental Medicine, Inha University School of Medicine, Incheon, Republic of Korea

## Abstract

This study investigated the effect of vitamin D supplementation in patients with dry eye syndrome (DES) refractory to conventional treatment with vitamin D deficiency. A total of 105 patients with DES refractory to conventional treatment and vitamin D deficiency that was treated with an intramuscular injection of cholecalciferol (200,000 IU). Serum 25-hydroxyvitamin D (25(OH)D) levels were measured. Eye discomfort was assessed using ocular surface disease index (OSDI) and visual analogue pain score (VAS). Tear break-up time (TBUT), fluorescein staining score (FSS), eyelid margin hyperemia, and tear secretion test were measured before treatment, and 2, 6, and 10 weeks after vitamin D supplementation. Mean serum 25(OH)D level was 10.52 ± 4.61 ng/mL. TBUT, and tear secretion test showed an improvement at 2 and 6 weeks after vitamin D supplementation compared to pretreatment values (p < 0.05 for all, paired t-test). Eyelid margin hyperemia and the severity of symptoms showed improvement at 2, 6, and 10 weeks after vitamin D supplementation (p < 0.05 for all). Compared to pre-treatment values, FSS, OSDI and VAS were decreased at 2 weeks (p < 0.05 for all). In conclusion, vitamin D supplementation is effective and useful in the treatment of patients with DES refractory to conventional treatment and with vitamin D deficiency.

Dry eye syndrome (DES) is a common ocular disease that is characterized by tear instability, ocular surface inflammation, and irritable eye symptoms^1^. DES is divided into two types: aqueous deficiency and evaporative type^2^. The reduction of tear secretion from lacrimal glands leads to aqueous deficiency DES^1^,^2^. Eyelid margin inflammation and meibomian gland dysfunction (MGD) have been suggested as major causes of evaporative type DES^2^. DES has been shown to cause inflammation of the ocular surface that is evidenced by increased levels of inflammatory cytokines in the tear fluid and corneal and conjunctival epithelia, and infiltration of CD4^+^ T cells into the conjunctiva^3^. The nuclear factor-κB (NF-κB) and mitogen-activated protein kinase (MAPK) pathways are activated in DES^4^. NF-κB is involved in signaling from the toll-like receptors (TLR) 2, 3, 4, 5 and 7, which are expressed in conjunctival, limbal, and corneal epithelial cells^5^. NF-κB is considered to be a prototypical proinflammatory signaling pathway^6^. NF-κB is stimulated by proinflammatory cytokines such as interleukin 1 (IL-1) and tumor necrosis factor α (TNFα)^7^. NF-κB regulates the expression of a wide variety of proinflammatory genes, including the genes for cytokines, chemokines, and adhesion molecules^6^. NF-κB plays a critical role in ocular surface inflammation and disease^5^. MAPKs, which are activated as a result of stimulation NF-κB, are known to stimulate the production of inflammatory cytokines and matrix metalloproteinases (MMPs)^8^. Th17 cells play an important role in DES^9^,^10^. Th17 cytokines are associated with disruption in corneal epithelial barrier function and can induce IL-6, transforming growth factor-β (TGF-β), IL-23 and IL-17A on the ocular surface of DES patients^4^,^10^. These pathogeneses contribute to the development of the ocular irritation associated with DES^11^. It has been suggested that the impact of severe DES on quality of life is similar to the impact of moderate to severe angina^11^. Patients with DES complain of chronic ocular fatigue and pain^12^.

Treatment for DES includes the use of artificial tears, anti-inflammatory drugs, autologous serum, and punctal occlusion^13^. Artificial tears contain carboxymethylcellulose or hyaluronate sodium and act as a lubricating agent at the ocular surface^13^. Artificial tears provide palliative relief of eye irritation in patients with aqueous tear deficiency, but do not treat the underlying inflammation or reverse conjunctival squamous metaplasia in chronic DES^14^. Conventional treatments using topical drugs and punctal occlusion are not effective in the treatment of chronic ocular pain in some patients with DES^15^.

Recently, vitamin D deficiency has been suggested to be a contributory factor in DES^16^,^17^. An association between DES and serum 25-hydroxyvitamin D (25(OH)D) concentration has been suggested^18^. It has been reported that vitamin D plays an immuno-modulatory role in innate and adaptive immunity^19^. Vitamin D and the vitamin D receptor (VDR) regulate several genes involved in inflammation, immunity, cellular proliferation, differentiation, and apoptosis^20^. However, the effect of vitamin D supplementation on DES has not been reported. In this study, we investigated the effect of vitamin D supplementation on the tear film and symptoms in the patients with DES that was refractory to conventional treatment.

## Materials and Methods

This observational study was performed in accordance with the tenets of the Declaration of Helsinki, and was reviewed and approved by the institutional review board/ethics committee of Hallym University Medical Center. The ethics committee/IRB waived the need for informed consent because this study is retrospective. We reviewed the medical charts from patients who had visited the Hallym University Kangnam Sacred Heart Hospital from June 2015 to March 2016. Patients with DES that was refractory to artificial tear treatment (hyaluronate sodium; New Hyaluni, Taejoon Pharm Co., Seoul) and liposic EDO (Bausch & Lomb, Gerhard Mann Gmbh) and with demonstrated vitamin D deficiency were included. Serum 25(OH)D concentration was measured. Patients with vitamin D deficiency or insufficiency were treated by an intramuscular injection of 200,000 IU cholecalciferol. Exclusion criteria were autoimmune diseases such as Sjogren’s syndrome or lupus syndrome; corneal surgery such as penetrating keratoplasty, corneal limbal allo-transplantation or corneal laceration repair; corneal diseases such as recurrent corneal erosion syndrome or keratoconus; and corneal opacity. Data was obtained pre-treatment, 2 weeks, 6 weeks, and 10 weeks after vitamin D supplementation.

Tear break-up time (TBUT), fluorescein staining score (FSS), and the Schirmer tear secretion test were used to evaluate the tear film. TBUT evaluation was performed in a dimly lit room. Fluorescein was placed in the lower conjunctival sac using a fluorescein strip (Haag-Streit, Köniz, Switzerland). The subjects were then asked to blink, and the time before the first defect appeared in the stained tear film was measured as TBUT. Fluorescein staining was performed as previously described^14^. After staining, corneal punctate erosion staining was recorded using the standardized Oxford grading system^21^. Schirmer’s test without topical anesthesia was performed to evaluate tear secretion in the patients^22^. Filter papers (Color Bar; EagleVision, Memphis, TN) were placed in the lateral canthus for 5 minutes. Readings were reported as millimeters of wetting.

Hyperemia and telangiectasia of the eyelid margin was graded as follows: 0 = none, 1 = mild, 2 = moderate, and 3 = severe. Conjunctivochalasis (CCH) was graded on the basis of the extent of inferior eyelid margin involvement as follows: 1 = single (temporal) location, 2 = two locations (nasal and temporal), and 3 = whole eyelid.

Eye discomfort was assessed by the ocular surface disease index (OSDI), visual analogue pain score (VAS), severity, and duration of symptoms. An OSDI questionnaire was used to quantify the dry eye symptoms. Subjects were asked questions regarding the symptoms of dry eye that they had experienced during a 1-week recall period; the OSDI questions comprised three different subscales: ocular symptoms, vision-related functions, and environmental triggers. Each answer was scored on a 4-point scale that ranged from zero (indicating no problems) to four (indicating a significant problem). Responses to all of the questions were combined to generate a composite OSDI score that ranged from 0 to 100, with higher OSDI scores indicating more severe symptoms^23^,^24^. Subjective symptoms were graded numerically using the VAS. The scale ranged from 0 (absence of pain) to 10 (maximal pain). The subjects were asked to describe their discomfort or pain using the VAS at each time point. Severity of symptoms was evaluated using a standard patient evaluation of eye dryness (SPEED) questionnaire^25^ as: 0 = no problems; 1 = tolerable-not perfect, but not uncomfortable; 2 = uncomfortable-irritating, but does not interfere with my day; 3 = bothersome-irritating and interferes with my day; and 4 = intolerable-unable to perform my daily tasks and requiring aggressive treatment. Duration of symptoms was evaluated as follows: 0, no symptom; 1, less than 1/3 of a day; 2, 1/3–1/2 of a day; and 3, 1/2 or more of a day.

### Statistics

All data are presented as mean and standard deviation. Paired-sample t-tests were used to compare the TBUT, FSS, eyelid margin hyperemia, Schirmer test, OSDI, VAS, and severity and duration of symptoms between pre-treatment, and 2, 6 and 10 weeks after vitamin D supplementation. SPSS version 23 for Windows (SPSS, Chicago, IL) was used for all analyses. P < 0.05 was considered to be statistically significant.

## Results

### Effect of vitamin D supplementation of dry eye syndrome

**Figure 1:**
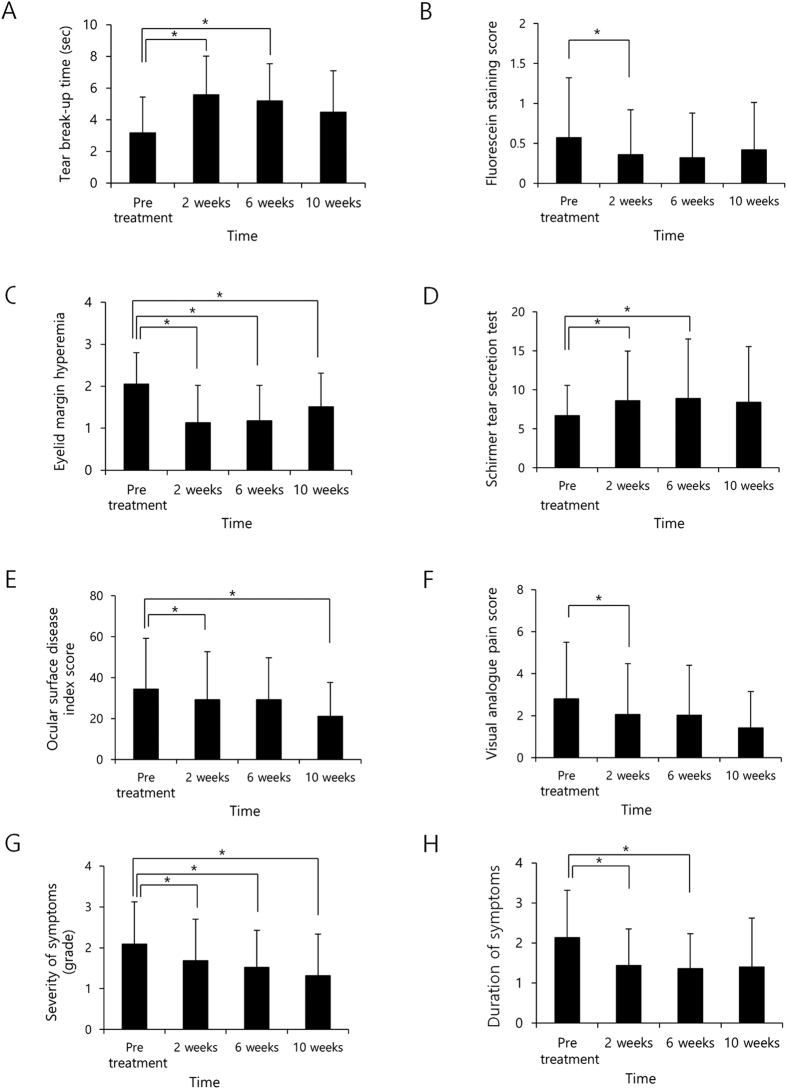
The effects of vitamin D supplementation on dry eye syndrome. (**A**) Tear break-up time was increased at 2 and 6 weeks but returned to pre-treatment levels after 10 weeks (p < 0.001, 0.001 and 0.066, respectively, paired t-test). (**B**) Fluorescein staining score decreased at 2 weeks and then increased at 6 and 10 weeks (p = 0.013, 0.088 and 0.826, respectively, paired t-test). (**C**) Hyperemia of the eyelid margin decreased at 2, 6, and 10 weeks (p < 0.001, <0.001 and 0.006). (**D**) Tear secretion by Schirmer test increased at 2 and 6 weeks but returned to pre-treatment levels at 10 weeks after vitamin D supplementation (p = 0.006, 0.015 and 0.140, respectively, paired t-test). (**E**) Ocular surface disease index (OSDI) decreased at 2, and 10 weeks (p = 0.046 and 0.004, respectively, paired t-test). (**F**) Visual analogue pain score (VAS) decreased at 2 weeks and then returned to pretreatment levels at 6 and 10 weeks (p = 0.005, 0.059 and 0.085, respectively, paired t-test). (**G**) Severity of symptoms decreased at 2, 6, and 10 weeks (p = 0.008, 0.001 and 0.045, respectively, paired t-test). (**H**) Score for duration of symptoms decreased at 2 and 6 weeks, and then returned to pre-treatment levels at 10 weeks after vitamin D supplementation (p = 0.005, 0.001 and 0.389, respectively, paired t-test). *statistically significant by paired t-test.

A total of 105 patients were included in the study (Table 1). The mean age of the patients was 58.21 ± 12.94 years. There were 21 men and 84 women. Mean serum 25(OH)D level was 10.52 ± 4.61 ng/mL. The effect of vitamin D supplementation on DES was assessed (Table 2, Fig. 1). TBUT was 3.16 ± 2.27 s at pre-treatment, increased to 5.58 ± 2.44 s after 2 weeks and to 5.19 ± 2.34 s after 6 weeks, before returning to the pre-treatment levels after 10 weeks (p < 0.001, 0.001 and 0.066, respectively, paired t-test). Pre-treatment FSS was 0.57 ± 0.75, 0.36 ± 0.56 after 2 weeks, 0.32 ± 0.56 after 6 weeks, and 0.42 ± 0.59 after 10 weeks (p = 0.013, 0.088 and 0.826, respectively, paired t-test). Hyperemia of the eyelid margin was 2.05 ± 0.75 at pre-treatment, and then decreased to 1.13 ± 0.89 after 2 weeks, 1.18 ± 0.84 after 6 weeks, and 1.51 ± 0.80 after 10 weeks (p < 0.001, <0.001 and 0.006). CCH was not significantly different throughout the course of the experimental periods. Tear secretion by Schirmer test was 6.69 ± 3.92 mm at pre-treatment, 8.64 ± 6.32 mm after 2 weeks, 8.92 ± 7.60 mm after 6 weeks, and 8.40 ± 7.16 mm after 10 weeks (p = 0.006, 0.015 and 0.140, respectively, paired t-test). OSDI was 34.33 ± 24.88 at pre-treatment, 29.25 ± 23.35 after 2 weeks, and 21.07 ± 16.52 after 10 weeks (p = 0.046 and 0.004, respectively, paired t-test). VAS was 2.80 ± 2.70 at pre-treatment, 2.05 ± 2.43 after 2 weeks, 2.02 ± 2.38 after 6 weeks, and 1.42 ± 1.73 after 10 weeks (p = 0.005, 0.059 and 0.085, respectively, paired t-test). Severity of symptoms was 2.09 ± 1.03 at pre-treatment, 1.68 ± 1.02 after 2 weeks, 1.52 ± 0.91 after 6 weeks and 1.32 ± 1.01 after 10 weeks (p = 0.008, 0.001 and 0.045, respectively, paired t-test). Symptom duration score was 2.14 ± 1.18 at pre-treatment, 1.68 ± 1.02 after 2 weeks, 1.52 ± 0.91 after 6 weeks, and 1.40 ± 1.22 after 10 weeks (p = 0.005, 0.001 and 0.389, respectively, paired t-test).

### Effects of vitamin D supplementation on DES according to gender

**Figure 2:**
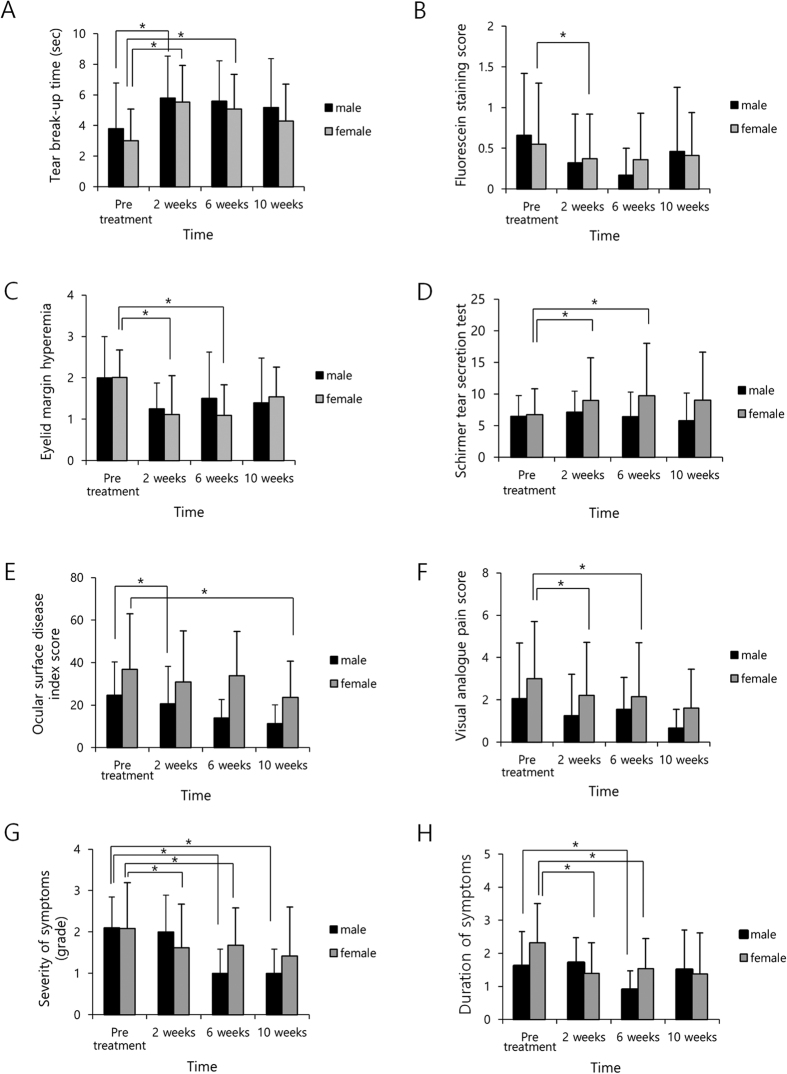
The effects of vitamin D supplementation on dry eye syndrome according to gender. (**A**) Tear break-up time (TBUT) in males was longer at 2 weeks compared to pre-treatment and that in female was longer at 2 and 6 weeks compared to pre-treatment (p = 0.041, <0.001 and <0.001, respectively, paired t-test). (**B**) Fluorescein staining score (FSS) in men was not different during the observational period but in women it was decreased at 2 weeks compared to pretreatment. (**C**) Hyperemia of the eyelid margin in men was lower at 6 and 10 weeks compared to pre-treatment and in women it was lower at 2 and 6 weeks compared to pre-treatment (p = 0.012, 0.030, <0.001 and <0.001, respectively, paired t-test). (**D**) Tear secretion in men was not different during the observational period, in women was increased at 2 and 6 weeks compared to pre-treatment (p = 0.009 and 0.011, respectively, paired t-test). (**E**) Ocular surface disease index (OSDI) score in men was lower at 6 weeks compared to pre-treatment; in women it was lower at 10 weeks compared to pre-treatment (p = 0.033 and 0.012, respectively, paired t-test). (**F**) Visual analogue pain score (VAS) in men did not different during the observational period; in women it was lower at 2 and 6 weeks compared to pre-treatment (p = 0.011 and 0.034, respectively, paired t-test). (**G**) Severity of symptoms in men was lower at 6 and 10 weeks; and in women it was lower at 2 and 6 weeks compared to pre-treatment (p = 0.043, 0.038, 0.017 and 0.014, respectively, paired t-test). (**H**) Duration of symptoms in men was lower at 6 weeks compared to pre-treatment; in women it was lower at 2 and 6 weeks compared to pre-treatment (p = 0.021, 0.004 and 0.021, respectively, paired t-test).

The effects of vitamin D supplementation on DES were analyzed according to gender (Fig. 2, Table 3). The TBUT in males was increased after 2 weeks compared to pre-treatment and in female it was increased after 2 and 6 weeks compared to pre-treatment (p = 0.041, <0.001 and <0.001, respectively, paired t-test). FSS in men showed no significant change over the entire observational period, but in women, it was decreased after 2 weeks compared to pretreatment. Hyperemia of the eyelid margin in men was lower at 6 weeks and at 10 weeks compared to pre-treatment and in women it was lower at 2 weeks and at 6 weeks compared to pre-treatment (p = 0.012, 0.030, <0.001 and <0.001, respectively, paired t-test). Tear secretion in men was not significantly different over the entire observational period, but in women it was higher at 2 weeks and 6 weeks compared to pre-treatment (p = 0.009 and 0.011, respectively, paired t-test). OSDI score in men was lower at 6 weeks compared to pre-treatment and in women it was lower at 10 weeks compared to pre-treatment (p = 0.033 and 0.012, respectively, paired t-test). VAS in men was not significantly different over the entire observational period, but in women it was lower at 2 weeks and at 6 weeks compared to pre-treatment (p = 0.011 and 0.034, respectively, paired t-test). The severity of symptoms in men was reduced at 6 and 10 weeks and in women it was lower at 2 and 6 weeks compared to pre-treatment (p = 0.043, 0.038, 0.017 and 0.014, respectively, paired t-test). Duration of symptoms in men was lower at 6 weeks compared to pre-treatment and in women it was lower at 2 and 6 weeks compared to pre-treatment (p = 0.021, 0.004 and 0.021, respectively, paired t-test).

### Effects of vitamin D supplementation on DES according to age group

**Figure 3:**
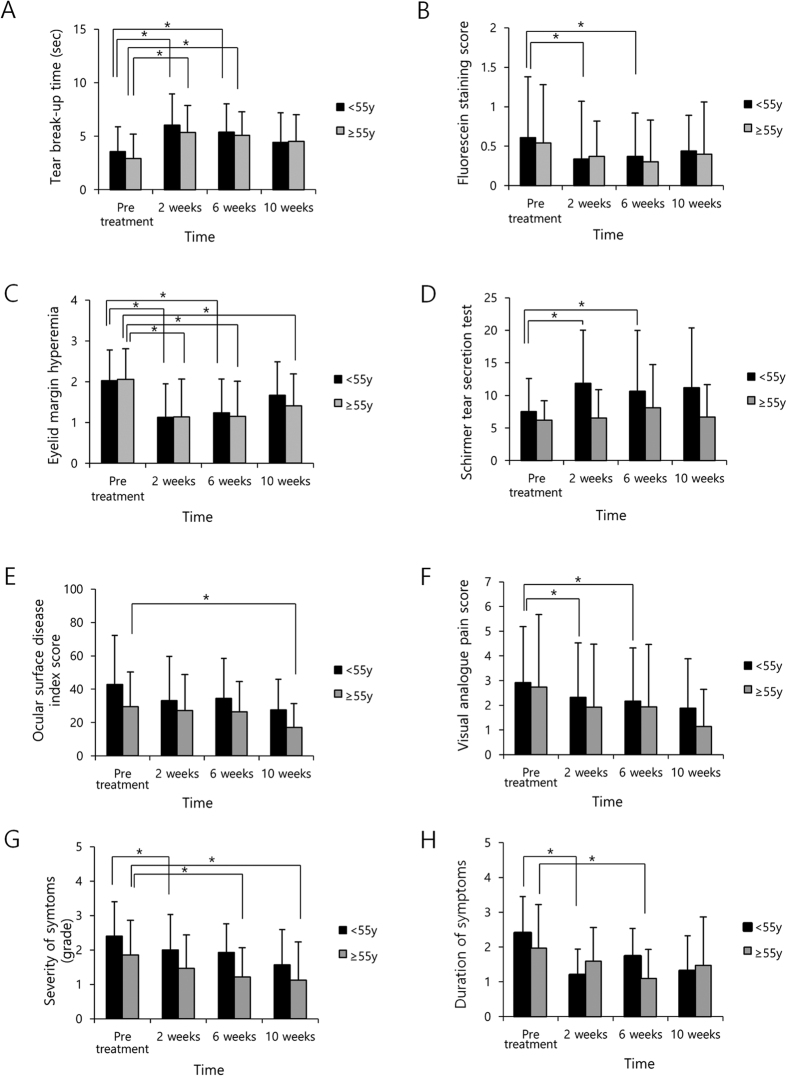
The effects of vitamin D supplementation on dry eye syndrome according to age group. Tear break-up time in both groups increased at 2 and 6 weeks compared to pre-treatment. TBUT in both groups was increased at 2 and 6 weeks compared to pre-treatment (p < 0.001 and 0.016 in the younger group and p < 0.001 and <0.001 in the older group, respectively, paired t-test). FSS in the younger group was lower at 2 and 6 weeks compared to pre-treatment (p = 0.016 and 0.035, respectively, paired t-test); results for the older group did not differ during the observational period. Hyperemia of the eyelid margin in the younger group was lower at 2 and 6 weeks compared to pre-treatment; in the older group it was decreased at 2, 6, and 10 weeks compared to pre-treatment (p < 0.001, 0.013, <0.001, <0.001 and 0.009, respectively, paired t-test). Tear secretion in the younger group was increased at 2 and 6 weeks compared to pre-treatment; no differences were observed in the older group (p = 0.014 and 0.040, respectively, paired t-test). OSDI score in the younger group did not change significantly during the observational period; in the older group it was lower at 10 weeks compared to pre-treatment (p = 0.010, paired t-test). VAS in the younger group was lower at 2 and 6 weeks compared to pre-treatment; no differences were found in the older group (p = 0.011 and 0.013, respectively, paired t-test). Severity of symptoms in the younger group was lower at 2 weeks compared to pre-treatment; in the older group they were lower at 6 and 10 weeks compared to pre-treatment (p = 0.013, 0.009 and 0.034, respectively, paired t-test). Duration of symptoms in the younger group was lower at 2 weeks compared to pre-treatment; in the older group they were lower at 6 weeks compared to pre-treatment (p = 0.007 and 0.011, respectively, paired t-test).

The effects of vitamin D supplementation on DES were analyzed according to age group (Fig. 3, Table 4). The subjects were divided into two groups by age: a younger group (age <55y) and an older group (age ≥55y). The TBUT in both the younger group and the older group was increased at 2 weeks and at 6 weeks when compared pre-treatment (p < 0.001 and 0.016 in the younger group and p < 0.001 and <0.001 in the older group, respectively, paired t-test). FSS in the younger group was lower at 2 and 6 weeks compared to pre-treatment (p = 0.016 and 0.035, respectively, paired t-test) but no significant difference was found for the older group throughout the entire observational period. Hyperemia of the eyelid margin in the younger group was lower at 2 and 6 weeks compared to that at pre-treatment; in the older group, it was lower at 2, 6, and 10 weeks compared to that at pre-treatment (p <0.001, 0.013, <0.001, <0.001 and 0.009, respectively, paired t-test). Tear secretion in the younger group was higher at 2 and 6 weeks compared to pre-treatment but in the older group it did not significantly differ during the observational period (p = 0.014 and 0.040, respectively, paired t-test). OSDI score in the younger group did not differ during the observational period but in the older group it was significantly lower at 10 weeks compared to pre-treatment (p = 0.010, paired t-test). VAS in the younger group was lower at 2 and 6 weeks compared to pre-treatment but in the older group it did not differ during the observational period (p = 0.011 and 0.013, respectively, paired t-test). The severity of symptoms in the younger group was lower after 2 weeks compared to pre-treatment and in the older group it was lower at 6 and 10 weeks compared to pre-treatment (p = 0.013, 0.009 and 0.034, respectively, paired t-test). The duration of symptoms in the younger group was lower at 2 weeks compared to pre-treatment and in the older group it was lower at 6 weeks compared to pre-treatment (p = 0.007 and 0.011, respectively, paired t-test).

## Discussion

DES has been reported to be associated with a variety of factors^2^. Recently, an association between DES and vitamin D deficiency has been suggested^16^,^26^. In this study, we examined the effect of vitamin D supplementation on DES that was refractory to conventional treatment. The vitamin D status of subjects was evaluated using serum 25(OH)D concentration. The concentration of 25(OH)D in the blood is regarded to be the best indicator of vitamin D status, because it is quantitatively related to the supply of vitamin D over the weeks preceding blood sample collection. The concentration of 25(OH)D reflects the supply of vitamin D from both the diet and from cutaneous synthesis under the influence of solar ultraviolet light^27^. In this study, intramuscular injection of vitamin D was used for the treatment of vitamin D deficiency. Two different forms of vitamin D supplementation can be used: oral and intramuscular injection^28^. Single intramuscular injection of vitamin D has been reported to be a safe and effective method for the increase and maintenance of serum 25(OH)D levels^29^.

In this study, vitamin D supplementation improved TBUT, FSS, eyelid margin hyperemia, and tear secretion. TBUT is the most frequently employed method for assessing tear instability^30^. Tear film instability is linked to tear hyperosmolarity, which is considered as a primary mechanism in the development of DES^31^. Tear film instability and hyperosmolarity induce ocular surface damage and initiate an inflammatory cascade that generates innate and adaptive immune responses in DES^9^. FSS and eyelid margin hyperemia are both associated with inflammation^30^,^32^. FSS has been used to assess ocular surface damage using sodium fluorescein although it is not specific for DES^30^. Eyelid margin hyperemia has been reported to contribute to DES and ocular surface disease^32^. Tear secretion is a major factor in the development of DES^32^. Reduced tear production results in tear instability and ocular surface damage^33^. Vitamin D supplementation was shown to promote tear secretion, reduce tear instability, and reduce inflammation of the ocular surface and eyelid margin. It has been suggested that DES is an autoimmune disease characterized by an immune and inflammatory processes that affect the ocular surface^34^. DES is an inflammatory disease that results from the activation of innate inflammatory pathways in resident ocular surface cells, as well as cytokines produced by recruited T helper (Th) cells^35^. In this study, vitamin D supplementation reduced FSS and hyperemia of the eyelid margin. FSS is an indicator of ocular surface inflammation. Eyelid margin hyperemia is related to meibomian gland dysfunction and eyelid inflammation, which is a major cause of evaporative type DES^32^,^36^. Vitamin D has extensive immunomodulatory effects^37^. The production of TNF-α and IFN-γ is significantly reduced by 1,25(OH)2 D3 through interference with NF-κB production^38^. 1,25(OH)2 D3 has immune regulatory effects on NK cell cytotoxicity, cytokine secretion, and the degranulation process as well as TLR4 expression^38^. Furthermore, 1,25(OH)2-Vitamin-D3 has been reported to attenuate Th17-related cytokines expression^39^. It has been reported that 1,25(OH)2 D3 ameliorated the inflammation of the colon and spleen by down-regulating the levels of Th 1 and Th17 cytokines^40^. The Th17 concentration in tears has been reported to be significantly increased in DES patients, with the concentration associated with the disease severity^10^. Another important cytokine in DES is IFN-γ^41^. IFN-γ is the signature cytokine from Th1 cells^32^. Increased IFN-γ concentration in tears of DES patients has been previously reported^41^. Vitamin D has the ability to suppress inflammatory cytokines such as TNF, IL-1, IFN- γ, and IL-2^37^.

In this study, vitamin D supplementation improved subjective symptoms including OSDI score, VAS score, severity, and duration of symptoms. A significant proportion of patients with DES complained of moderate or greater ocular pain intensity^15^. DES patients reported more frequent chronic pain syndromes^12^,^42^. The ocular symptoms in DES patients have been suggested to be a result of neuropathic pain^43^. An inverse correlation has been observed between vitamin D and OSDI scores or dendritic cell density^44^. Vitamin D deficiency is associated with chronic pain and central hypersensitivity in patients with chronic pain^45^,^46^. Vitamin D supplementation has been reported to improve pain, sleep, and quality of life in chronic pain patients^47^. Singman *et al*. reported a case of putative corneal neuralgia accompanying hypovitaminosis D and responding to vitamin D supplementation^48^. The effects of vitamin D supplementation returned to pretreatment levels after 10 weeks in this study. Therefore, vitamin D supplementation using intramuscular injection of cholecalciferol (200,000 IU) should be performed every 10 weeks for the management of DES.

TBUT increased in both male and female patients, whereas FSS and tear secretion was only improved in women. The effect of vitamin D has been reported to differ according to gender^49^,^50^. Vitamin D is an important factor in estrogen biosynthesis and estrogen signaling, and has anti-estrogenic activity^51^,^52^. Synthesis and bioavailability of vitamin D change according to age^47^. In this study, TBUT was increased in both groups after 2 and 6 weeks compared to pre-treatment. The FSS and tear secretion in the younger group improved at 2 and 6 weeks compared to pre-treatment but did not differ in the older group. Reduction of eyelid margin hyperemia was prolonged in the older group compared to the younger group.

## Conclusions

In summary, vitamin D supplementation promoted tear secretion, reduced tear instability, and reduced inflammation at the ocular surface and eyelid margin. Furthermore, vitamin D supplementation improved the symptoms of DES. In conclusion, vitamin D supplementation is an effective and useful treatment for patients with DES that is refractory to conventional treatment.

## Additional Information

**How to cite this article**: Bae, S. H. *et al*. Vitamin D Supplementation for Patients with Dry Eye Syndrome Refractory to Conventional Treatment. *Sci. Rep.* **6**, 33083; doi: 10.1038/srep33083 (2016).

## Figures and Tables

**Table 1:** Demographic data of subjects.

	Total subjects
N	105
Age	58.21 ± 12.94 years
Gender (male: female)	21:84
Serum 25 (OH)D levels	10.52 ± 4.61 ng/mL

**Table 2:** The effect of vitamin D supplementation on dry eye syndrome.

	Pre-treatment (n = 105)	After vitamin D supplementation					
		2 weeks (n = 78)		6 weeks (n = 54)		10 weeks (n = 49)	
	Mean ± SD	Mean ± SD	p-value	Mean ± SD	p-value	Mean ± SD	p-value
TBUT (sec)	3.16 ± 2.27	5.58 ± 2.44	<0.001*	5.19 ± 2.34	<0.001*	4.49 ± 2.60	0.066
FSS (grade)	0.57 ± 0.75	0.36 ± 0.56	0.013*	0.32 ± 0.56	0.088	0.42 ± 0.59	0.826
Hyperemia of eyelid margin (grade)	2.05 ± 0.75	1.13 ± 0.89	<0.001*	1.18 ± 0.84	<0.001*	1.51 ± 0.80	0.006*
CCH (grade)	0.40 ± 0.77	0.36 ± 0.73	0.884	0.28 ± 0.74	0.537	0.31 ± 0.54	1.000
Schirmer tear secretion test (mm)	6.69 ± 3.92	8.64 ± 6.32	0.006*	8.92 ± 7.60	0.015*	8.40 ± 7.16	0.140
OSDI score	34.39 ± 24.88	29.25 ± 23.35	0.046*	29.20 ± 20.44	0.136	21.07 ± 16.52	0.004*
VAS score	2.80 ± 2.70	2.05 ± 2.43	0.005*	2.02 ± 2.38	0.059	1.42 ± 1.73	0.085
Severity of symptoms (grade)	2.09 ± 1.03	1.68 ± 1.02	0.008*	1.52 ± 0.91	0.001*	1.32 ± 1.01	0.045*
Duration of symptoms (grade)	2.14 ± 1.18	1.44 ± 0.91	0.005*	1.36 ± 0.87	0.001*	1.40 ± 1.22	0.389

TBUT = tear break-up time; FSS = fluorescein staining score; CCH = conjunctivochalasis; OSDI = ocular surface disease index; VAS = visual analogue scale; **p < 0.05 by paired t-test compared to pre-treatment.

**Table 3:** The effect of vitamin D supplementation on dry eye syndrome according to gender.

male:female (N)		Pre-treatment	After vitamin D supplementation					
			2 weeks		6 weeks		10 weeks	
		21:84	14:84		12:42		11:38	
		Mean ± SD	Mean ± SD	p-value	Mean ± SD	p-value	Mean ± SD	p-value
TBUT (sec)	M	3.80 ± 2.98	5.79 ± 2.75	0.041*	5.58 ± 2.64	0.147	5.18 ± 3.19	0.346
	F	3.01 ± 2.06	5.53 ± 2.39	<0.001*	5.07 ± 2.27	<0.001*	4.29 ± 2.42	0.123
FSS (grade)	M	0.66 ± 0.76	0.32 ± 0.60	0.267	0.17 ± 0.33	0.213	0.46 ± 0.79	0.279
	F	0.55 ± 0.75	0.37 ± 0.55	0.026*	0.36 ± 0.57	0.202	0.41 ± 0.53	0.909
Hyperemia of eyelid margin (grade)	M	2.00 ± 1.00	1.25 ± 0.62	0.137	1.50 ± 1.12	0.012*	1.40 ± 1.08	0.030*
	F	2.01 ± 0.66	1.11 ± 0.94	<0.001*	1.09 ± 0.74	<0.001*	1.54 ± 0.72	0.090
CCH (grade)	M	0.25 ± 0.45	0.29 ± 0.49	0.391	0.09 ± 0.30	0.351	0.00 ± 0.00	
	F	0.43 ± 0.83	0.37 ± 0.76	0.879	0.36 ± 0.86	0.331	0.38 ± 0.57	1.000
Schirmer tear secretion test (mm)	M	6.50 ± 3.28	7.15 ± 3.31	0.401	6.42 ± 3.87	0.735	5.78 ± 4.38	0.770
	F	6.73 ± 4.08	8.97 ± 6.77	0.009*	9.71 ± 8.33	0.011*	9.03 ± 7.59	0.104
OSDI score	M	24.54 ± 15.77	20.60 ± 17.54	0.139	13.97 ± 8.66	0.033*	11.27 ± 8.77	0.194
	F	36.79 ± 26.15	30.81 ± 24.04	0.099	33.78 ± 20.79	0.489	23.51 ± 17.16	0.012*
VAS score	M	2.05 ± 2.64	1.25 ± 1.96	0.252	1.55 ± 1.51	0.796	0.67 ± 0.87	0.384
	F	3.00 ± 2.70	2.21 ± 2.50	0.011*	2.14 ± 2.56	0.034*	1.61 ± 1.84	0.131
Severity of symptoms (grade)	M	2.10 ± 0.74	2.00 ± 0.89	0.175	1.00 ± 0.58	0.043*	1.00 ± 0.58	0.038*
	F	2.08 ± 1.11	1.62 ± 1.05	0.017*	1.68 ± 0.90	0.014*	1.42 ± 1.18	0.356
Duration of symptoms (grade)	M	1.62 ± 1.04	1.71 ± 0.76	1.000	0.90 ± 0.57	0.021*	1.50 ± 1.20	0.749
	F	2.32 ± 1.19	1.39 ± 0.93	0.004*	1.54 ± 0.91	0.021*	1.37 ± 1.25	0.356

TBUT = tear break-up time; FSS = fluorescein staining score; CCH = conjunctivochalasis; OSDI = ocular surface disease index; VAS = visual analogue scale; *p < 0.05 by paired t-test compared to pre-treatment.

**Table 4:** The effect of intramuscular vitamin D injection on dry eye syndrome according to age.

Younger group: older group (N)		Pre-treatment 39:66	After vitamin D supplementation					
			2 weeks 25:53		6 weeks 19:35		10 weeks 18:31	
		Mean ± SD	Mean ± SD	p-value	Mean ± SD	p-value	Mean ± SD	p-value
TBUT (sec)	<55y	3.56 ± 2.35	6.04 ± 2.92	<0.001*	5.37 ± 2.65	0.016*	4.44 ± 2.75	0.393
	≥55y	2.92 ± 2.21	5.36 ± 2.18	<0.001*	5.09 ± 2.19	<0.001*	4.52 ± 2.55	0.098
FSS (grade)	<55y	0.61 ± 0.77	0.34 ± 0.73	0.016*	0.37 ± 0.55	0.035*	0.44 ± 0.45	0.653
	≥55y	0.54 ± 0.74	0.37 ± 0.45	0.171	0.30 ± 0.53	0.408	0.40 ± 0.66	0.434
Hyperemia of eyelid margin (grade)	<55y	2.03 ± 0.75	1.13 ± 0.82	<0.001*	1.24 ± 0.83	0.013*	1.67 ± 0.82	0.339
	≥55y	2.06 ± 0.75	1.14 ± 0.93	<0.001*	1.15 ± 0.86	<0.001*	1.41 ± 0.78	0.009*
CCH (grade)	<55y	0.10 ± 0.30	0.00 ± 0.00	0.337	0.00 ± 0.00	0.341	0.23 ± 0.44	1.000
	≥55y	0.57 ± 0.90	0.57 ± 0.85	0.880	0.43 ± 0.90	0.751	0.37 ± 0.60	1.000
Schirmer tear secretion test (mm)	<55y	7.53 ± 5.08	11.88 ± 8.16	0.014*	10.63 ± 9.34	0.040*	11.17 ± 9.21	0.050
	≥55y	6.19 ± 2.97	6.56 ± 4.32	0.198	8.12 ± 6.64	0.122	6.69 ± 4.99	0.886
OSDI index	<55y	42.92 ± 29.27	33.16 ± 26.44	0.082	34.36 ± 24.04	0.133	27.58 ± 18.22	0.148
	≥55y	29.54 ± 20.72	27.17 ± 21.55	0.337	26.48 ± 18.05	0.557	17.11 ± 14.31	0.010*
VAS score	<55y	2.92 ± 2.27	2.32 ± 2.21	0.011*	2.17 ± 2.15	0.013*	1.88 ± 2.00	0.348
	≥55y	2.73 ± 2.94	1.92 ± 2.55	0.058	1.94 ± 2.52	0.409	1.14 ± 1.51	0.154
Severity of symptoms (grade)	<55y	2.40 ± 1.00	2.00 ± 1.03	0.013*	1.93 ± 0.83	0.067	1.57 ± 1.02	0.477
	≥55y	1.85 ± 1.01	1.46 ± 0.98	0.076	1.21 ± 0.86	0.009*	1.12 ± 1.11	0.034*
Duration of symptoms (grade)	<55y	2.40 ± 1.05	1.19 ± 0.75	0.007*	1.73 ± 0.80	0.052	1.31 ± 1.01	0.208
	≥55y	1.97 ± 1.25	1.59 ± 0.97	0.175	1.10 ± 0.83	0.011*	1.47 ± 1.39	1.000

TBUT = tear break-up time; FSS = fluorescein staining score; CCH = conjunctivochalasis; OSDI = ocular surface disease index; VAS = visual analogue scale; **p < 0.05 by paired t-test compared to pre-treatment.
